# Der Übergang vom Kindergarten in die Grundschule – eine Orientierung zum Stand der empirischen Forschung

**DOI:** 10.1007/s42278-023-00171-4

**Published:** 2023-05-30

**Authors:** Daniel Mays, Carolin Quenzer-Alfred, Franka Metzner-Guczka, Holger Zielemanns, Lisa Tölle, Vivien Soyka, Leonie Krol, Michelle Lok-Yan Wichmann

**Affiliations:** Professur für Erziehungswissenschaft mit dem Schwerpunkt Förderpädagogik („Emotionale und soziale Entwicklung“), Universität Siegen, Adolf-Reichwein-Str. 2, 57068 Siegen, Deutschland

**Keywords:** Übergang, Kindergarten, Grundschule, Einschulung, Schuleintritt, Transition, Preschool, Elementary school, School enrollment

## Abstract

Die vorliegende Literaturübersicht hat das Ziel, vorhandene internationale empirische Studien zum Übergang vom Kindergarten in ein staatliches oder privates Schulsystem zusammenzufassen. Um den Forschungsstand zusammenzufassen, wurde sich an den Methoden eines Scoping-Reviews orientiert. Dazu wurden neun wissenschaftliche Datenbanken durchsucht. In einem mehrstufigen Selektionsprozess wurden aus insgesamt *k* = 6492 Funden über den Abgleich mit sieben a priori festgelegten Einschlusskriterien *k* = 47 deutsch- und englischsprachige Studien zur deskriptiven Analyse in das Scoping-Review eingeschlossen. Die in den Literaturüberblick eingeschlossenen Studien wurden vorrangig in Deutschland (33 %) und in den USA (29 %) durchgeführt. Sechs wesentliche Forschungsschwerpunkte konnten in den sowohl quantitativen, qualitativen und Mixed-Methods-Forschungsdesigns herausgearbeitet werden. Dabei konzentrieren sich die meisten Studien auf die Entwicklung und explorative Bewertung von Fördermöglichkeiten am Übergang als auch strukturierte Programme, die konzeptionell in Kindergarten und Grundschule verankert wurden. Zwei weitere Schwerpunkte liegen in den eingeschlossenen Studien auf der explorativen Betrachtung der Entwicklung von Leistungen, Kompetenzen und Engagement während des Übergangs, sowie auf Sozial- und Problemverhalten im Übergangsprozess und allgemeinen sozialen Herausforderungen im Zuge der notwendigen Anpassung an das System Grundschule. Darüber hinaus werden Lücken und Nutzen von Übergangspraktiken, Unterschiede zwischen Kindergarten und Grundschule als auch in einigen wenigen Studien das Thema „Inklusion und Übergangsgestaltung“ beleuchtet.

## Abstract (en)

This review aims to summarize existing international empirical studies on the transition from kindergarten to a state or private school system. In order to summarize the state of research, the methods of a scoping review were followed. For this purpose, nine scientific databases were searched. In a multistage selection process, *k* = 47 German- and English-language studies were included in the scoping review for descriptive analysis from a total of *k* = 6492 findings via matching with seven a priori defined inclusion criteria. The included studies were primarily conducted in Germany (33%) and the United States (29%). Six main research themes could be identified in the both quantitative, qualitative, and mixed-methods research designs. Most of the studies focused on the development and exploratory evaluation of support options during transition as well as structured programs that were conceptually embedded in preschool and elementary schools. Two additional areas of focus in the included studies are the exploratory examination of the development of performance, skills, and engagement during transition, as well as social and problem behavior during the transition process and general social challenges in the course of the necessary adjustment to the elementary school system. Furthermore, gaps and benefits of transition practices, differences between preschool and elementary school, as well as—in a few studies—the topic “inclusion and transition design” are highlighted.

## Einleitung

### Theoretischer Rahmen

Bildungsübergänge sind entscheidende Wandlungsprozesse in der Biografie von Schülerinnen und Schülern, die mit vielschichtigen Veränderungen der kindlichen Lebenssituation einhergehen und einen entscheidenden Einfluss auf Identitätsentwicklung, Leistungsfähigkeit und Schulerfolg haben (Griebel und Niesel 2003; Hattie 2013).

Von besonderem Interesse der auf das Schulsystem bezogenen Übergangsforschung ist dabei der Übergang zwischen dem elementarpädagogischen Vorschulbereich zum Pflichtschulbereich, den die meisten schulpflichtigen Kinder passieren müssen und als entscheidender Abschnitt in der frühen Bildungsbiografie von Kindern diskutiert wird (Oehlmann et al. 2011). Vor dem Hintergrund der Covid-19-Pandemie rückt das Thema nun noch einmal verstärkt in den Vordergrund: in dem im November 2022 veröffentlichten RKI-Abschlussbericht der Corona-Kita-Studie berichten Kita-Leitungen von pandemiebedingt stark gestiegenen vorschulischen Förderbedarfen. 58 % der befragten Kita-Leitungen berichten von gestiegenen Förderbedarfen der (Vorschul‑)Kinder im Bereich der emotionalen und sozialen Entwicklung, 43 % der Leitungen verweisen auf gestiegene Förderbedarfe in der sprachlichen Entwicklung und 46 % in der motorischen Entwicklung. Noch besorgniserregendere Rückmeldungen kamen aus Einrichtungen mit einem höheren Anteil an Kindern aus benachteiligten Verhältnissen (Kuger et al. 2022).

Sowohl der nationale als auch der internationale Forschungsstand zum Übergang in die Grundschule zeichnet ein uneinheitliches Bild zu möglichen positiven wie negativen Auswirkungen des Schuleintritts auf die kindliche Entwicklung. Ein großer Teil der Kinder scheint den Übergang schlussendlich gut bewältigen zu können – auch wenn im Verlaufe dieser Phase ein Kind möglicherweise vereinzelte Irritationen durchlebt und Herausforderungen bewältigt (Jindal-Snape und Foggie 2008).

Auf der Verhaltensebene des Kindes können sich belastende Dynamiken im Transitionsverlauf im ungünstigen Fall jedoch auch bei manchen Kindern manifestieren und beispielsweise in Stresssymptomen, wie Wut oder Depression, Schüchternheit, emotionalen und sozialen Anpassungsproblemen bei der Bewältigung des Alltags und der Verantwortung oder in mangelnder Akzeptanz innerhalb ihrer Peergruppe, mangelnder sozialer Integration und mangelnder Fähigkeit, neue Freunde zu finden, äußern (Beelmann 2006; Grotz 2005; Griebel und Niesel 2004).

Für den US-amerikanischen Raum hat das National Center for Early Development and Learning (NCDEL 1996) in ihrer Umfrage zu Übergangspraktiken bei rund zwei Fünfteln der Kinder, die in die Schule gehen, vergleichbare Übergangsprobleme beobachtet (Pianta und Cox 1999; Rimm-Kaufmann und Pianta 2000). In Polen zeigten 50 % der untersuchten Kinder „soziale Anpassungsprobleme“ (Kienig 2002). Verschiedene Phasen des Übergangs für Kinder zu Schulbeginn sind nachweislich mit Stress verbunden (Fabian 2002; Griebel und Niesel 2002, 2003, 2004; Margetts 2002; Parent et al. 2019). Eine zusammenfassende und altersstufenübergreifende Analyse von 181 Studien konnte zudem zeigen, dass misslungene Übergänge sich negativ auf den schulischen Erfolg des Kindes auswirken können, aber positive Entwicklungen z. B. über eine frühe Anbahnung von neuen Freundschaften in der neuen Lernumgebung unterstützt werden können (Hattie 2013).

Inzwischen offenbaren Daten aus empirischen Studien, dass Schulanfängerinnen und -anfänger, die einen erfolgreichen Schulanfang und damit Übergang in das staatlich verpflichtende Bildungssystem erleben, später eher schulische, gesellschaftliche und berufliche Erfolge zeigen als diejenigen Kinder, die in der ersten Schulzeit Schwierigkeiten auf unterschiedlichen Ebenen zeigten (Gutman et al. 2003; LoCasale-Crouch et al. 2008; Shields 2009).

In weiteren Studien konnte aufgezeigt werden, dass im letzten Kitabesuchsjahr vorhandene (vor‑)schulische Kompetenzen den zukünftigen Schulerfolg und auch Schulleistungen anteilig vorhersagen können. Genannt seien hier z. B. die EPPE-Studie (Sylva et al. 2003) oder die SPEEL-Studie (Moyles et al. 2002).

In einer Längsschnittstudie zur Früherkennung von Rechenschwächen (Krajewski 2008) wird berichtet, dass die mathematischen Leistungen von Kindern in der vierten Klasse im letzten Vorschuljahr anhand der Zahlenkompetenzen oftmals vorhergesagt werden können. Neben dem numerischen Vorwissen gelten zudem auch die Lesekompetenz, die Entwicklung schriftsprachlicher Kompetenzen und die phonologische Bewusstheit als Prädiktoren für zukünftige Schulleistungen (z. B. Schneider und Näslund 1999; Krajewski 2008).

Transitionen bedingen häufig Veränderungen in sehr kurzer Zeit in unterschiedlichen Kompetenzbereichen und auf der individuellen, der interaktionalen und der kontextuellen Ebene der Lebens- und Lernwelten, deren positive Bewältigung entscheidend für Teilhabechancen im Bildungssystem und für das Gelingen der späteren Lebensführung sein kann (Griebel und Niesel 2011; Gisbert 2003; Rabe-Kleberg 2011). Zu konstatieren ist jedoch, dass die Forschungslage im Kontext der Übergangsforschung vom Kindergarten in die Grundschule gerade wegen und/oder trotz ihrer Popularität insgesamt als unübersichtlich einzustufen ist und keine aggregierende Forschungsarbeiten vorliegen.

Welche Evidenz aus dem (inter‑)nationalen Raum zum Übergang Kita-Grundschule vorliegt, ist Gegenstand dieser Übersichtsarbeit.

### Ziele und Forschungsfragen

Primäres Ziel des vorliegenden Beitrags ist die Zusammenfassung und Beschreibung empirisch fundierter Erkenntnisse aus dem nationalen und internationalen Raum zum Übergang vom Kindergarten in die Grundschule. Mit Blick auf die möglichen vielfältigen psychosozialen Folgen eines nicht gut bewältigten Übergangs (vgl. Beelmann 2006; Grotz 2005; Griebel und Niesel 2004) soll dabei auch die emotionale und soziale Entwicklung der Kinder in den Blick genommen werden. Darüber hinaus sollen als sekundäres Ziel nicht hinreichend beleuchtete Forschungsfelder identifiziert werden. Zu diesem Zweck werden die Forschungsschwerpunkte, untersuchten Fragestellungen sowie die Methodik identifizierter Originalstudien beschrieben sowie deren Befunde zu relevanten Faktoren (z. B. auf individueller oder kontextueller Ebene) für diesen Übergang in einer Datensynthese zusammengefasst. Mit der Zusammenfassung der Forschungsliteratur sollen die folgenden grundlegenden Fragen beantwortet werden:

- Welche empirischen Erkenntnisse aus dem (inter‑)nationalen Raum wurden mit welchen inhaltlichen Forschungszielen bisher zum Übergang vom Kindergarten in die Grundschule veröffentlicht?
- Was sind die wesentlichen Forschungsmethoden, die in den Artikeln des Literaturüberblicks zum Tragen kommen?
- Welches sind die wesentlichen Einschränkungen und Möglichkeiten für weitere Studien zur Übergangsforschung an der Schnittstelle Kita – Grundschule im Rahmen der Übersichtsarbeit?

## Methoden

Um die empirische Forschungslage zum Übergang vom Kindergarten in die Grundschule zusammenzufassen wurde sich an der Methodik eines systematischen Scoping-Reviews (Arksey und O’Malley 2005) orientiert, um über diese streng systematische und transparente Methodik möglichst alle relevanten Studien in dem fokussierten Themenbereich zusammen tragen zu können. Scoping-Reviews dienen dazu den potenziellen Umfang und Reichweite der verfügbaren Forschungsliteratur vorläufig zu identifizieren, Orientierung zu bieten und somit Art und Umfang der Forschungsergebnisse zu ermitteln (Grant und Booth 2009; Biondi-Zoccai 2016). Damit ist das Scoping-Review von anderen Review-Typen wie dem klassischen systematischen Review abzugrenzen, welche sich mit relativ präzisen Fragestellungen wie z. B. der Wirksamkeit von Interventionen und genau definierten Outcome-Variablen beschäftigen.

Der vorliegende Beitrag wurde unter Berücksichtigung von Leitlinien und Empfehlungen für die Durchführung und Auswertung systematischer Literaturübersichten in der Psychologie und Erziehungswissenschaft (z. B. Higgins et al. 2019; Zawacki-Richter et al. 2020; Moher et al. 2009) umgesetzt. Auf dieser Basis wurde der vorliegenden Übersichtsarbeit ein etabliertes Protokoll mit fünf Schlüsselphasen für einen mehrstufigen Selektionsprozess zugrunde gelegt: (1) Identifizierung der Forschungsfrage, (2) Identifizierung relevanter Studien mithilfe eines festgelegten Suchstrings in neun wissenschaftlichen Fachdatenbanken, (3) Auswahl der Studien auf Basis festgelegter Einschlusskriterien, (4) Erfassung und Codierung der Daten und (5) Zusammenstellung, Zusammenfassung und Berichterstattung der Ergebnisse (Arksey und O’Malley 2005).

### Suchstrategien und Einschlusskriterien

Zur Identifikation von relevanten Publikationen wurden die elektronischen Datenbanken FIS Bildung Literaturdatenbank bzw. Fachportal Pädagogik, Web of Science, Medline, PsycINFO, CINAHL, BIOSIS, ERIC, ASSIA und PSYNDEX nach in Fachzeitschriften veröffentlichten deutsch- oder englischsprachigen Originalarbeiten durchsucht. In Abhängigkeit der Suchoptionen in den unterschiedlichen Datenbanken wurden für die Suche angepasste Suchstrings aus den zuvor getesteten, kombinierten und teilweise trunkierten Suchbegriffen auf Deutsch und Englisch wie z. B. „transition*“, „school*“, kindergart*, „preschool*“, „Grundsch*“, „Uebergang“ und „Schul*“ verwendet. Um den Publikationsbias zu reduzieren, wurden zusätzlich Literaturverzeichnisse von relevanten Studien, thematisch passende Übersichtsarbeiten sowie die Suchmaschine Google Scholar manuell durchsucht. Für das Screening und die Auswahl relevanter Publikationen wurden sieben Einschlusskriterien (EK) a priori festgelegt. Für den Einschluss in die Literaturübersicht mussten die Studien

- Kinder im letzten Jahr vor Übergang ins formale Schulsystem^1^ bzw. im ersten Jahr im formalen Schulsystem (in‑)direkt (EK 1),
- einen Bildungsübergang vom Kindergarten ins formale Schulsystem (EK 2) und
- relevante Faktoren im Kontext des Übergangs Kindergarten-Grundschule (z. B. intraindividuell, interindividuell, kontextuell) untersucht haben (EK 3),
- Daten an den Kindern oder über die Kinder hinsichtlich ihres Bildungsübergangs aus Perspektive der Kinder selbst, Eltern oder Erzieherinnen und Erzieher bzw. Lehrerinnen und Lehrer erhoben haben (EK 4),
- ein qualitatives, quantitatives oder Mixed-Methods Studiendesign aufweisen (EK 5),
- in einer Fachzeitschrift mit Peer-Review (EK 6) sowie
- auf Deutsch oder Englisch publiziert sein (EK 7).

### Selektion, Kodierung und Datenextraktion

Von *k* = 6492 über die Datenbanksuchen identifizierten Publikationen schlossen zwei unabhängige Reviewerinnen und Reviewer nach dem Screening von Titel und Abstract und dem Abzug der Duplikate *k* = 106 Publikationen für die Volltextbewertung ein. Nach der Bewertung der Volltexte wurden *k* = 47 Publikationen für den Einschluss in die Datenextraktion und -synthese ausgewählt. Die Publikationen wurden in beiden Selektionsschritten von den beiden unabhängigen Reviewerinnen und Reviewern hinsichtlich ihrer Übereinstimmung mit den o. g. Einschlusskriterien bewertet. Jedes Einschlusskriterium wurde dazu mit „Ja“ (ist gegeben), „Nein“ (ist nicht gegeben) oder „Unklar“ (fehlende oder nicht eindeutige Information) eingeschätzt. Studien, die mindestens ein Einschlusskriterium nicht erfüllten, wurden ausgeschlossen. Nicht übereinstimmende Bewertungen wurden diskutiert, bis ein Konsens erreicht wurde.

Aus den *k* = 47 eingeschlossenen Publikationen wurden grundlegende Informationen mit Hilfe einer auf die Forschungsfrage angepassten Kodierstrategie extrahiert (vgl. Evidence for Policy and Practice; EPPI 2001), z. B. Name der Erstautorin bzw. des Erstautors, Publikationsjahr und -ort, Titel der Arbeit. Zusätzlich wurden folgende Merkmale zur deskriptiven Synthese aller Studien systematisch erfasst: Gegenstand der Studie bzw. Forschungsfrage, Forschungsziel, Studientyp bzw. -design und Datenerhebungsmethode, Stichprobengröße, Schulform, Alter der Stichprobe und Perspektive, Bildungssysteme, ggfs. Förderbedarf, zentrale Ergebnisse und untersuchte Variablen.

## Ergebnisse

Im Folgenden werden die Charakteristika der *k* = 47 eingeschlossenen Studien beschrieben (s. Tab. 1).

**Table 1:** 

#	Autorschaft/-en (Publikationsjahr) [Erhebungsland]	Gegenstand und Ziele der Studie	Studientyp (Datenerhebung) [Design] {Perspektive}	Stichprobe Kinder: *N* (% weiblich) [sonst. Charakteristika]	Alter in Jahren: Mittelwert (SD) [Range]
1	Ahnert und Harwardt (2008) [Deutschland]	Zusammenhang zwischen Beziehungserfahrungen (zu Erzieherin im Kindergarten, Mutter) und Freude an Spiel bzw. Lernen, Anstrengungsbereitschaft sowie Lernmotivation in Vorschule/nach Schulbeginn	Quantitativ (Einschätzungsfragebogen, Leistungsstanderhebung) [N. A.] {FuF, EuE, Eltern, Kinder}	100 (53) [N. A.]	6,1 (N. A.) [N. A.]
2	Alatalo et al. (2016) [Schweden]	Erfahrungen von LuL bzgl. des Übergangs zwischen Vor- und Grundschule	Mixed-Method (Selbstentwickelter Fragebogen, halbstrukturierte Interviews) [N. A.] {Vorschul-LuL, Grundschul-LuL}	0 (–) [–]	–
		Berücksichtigung von förderlichen Faktoren für die Kontinuität und das langfristige Lernen der Kinder in den Zielbereichen des schwedischen Lehrplans			
3	Arndt et al. (2013) [Deutschland]	Erfassung der Perspektiven von Eltern und Fachkräften bzgl. der kindlichen Lernprozesse während des Übergangs zwischen Kindergarten und Grundschule	Qualitativ (Episodische Interviews) [Längsschnitt; 3 Erhebungszeitpunkte] {Kinder, Eltern, EuE, LuL}	19 (N. A.) [sozioökonomisch benachteiligte Familien]	N. A. (N. A.) [N. A.]
		Untersuchung des Zusammenhangs zwischen Lern- und Entwicklungsprozessen während des Übergangs			
4	Babić (2017) [Kroatien]	Erfassung kindlicher Perspektiven bzgl. Kindergarten und Schule zur Beleuchtung deren Verständnisses von „Kindheit“ und „Kindern“	Qualitativ (Halbstrukturierte Paarinterviews) [N. A.] {Kinder}	20 (60) [N. A]	6,4 (N. A.) [N. A.]
5	Benner et al. (2017) [N. A.]	Prüfung des Zusammenhangs zwischen kindlichen bildungsbezogenen und sozioemotionalen Outcomes nach Schuleintritt und der Inanspruchnahme von Nachhaltigkeitspraktiken (inkl. Kontrolle für familiäre und schulische Selektionsmechanismen, Prüfung eines moderierenden Effekts der Mechanismen)	Mixed-Method (Befragung, Interviews) [Längsschnitt; 4 Erhebungszeitpunkte] {EuE, Kinder}	5050 (50) [N. A.]	6,2 (N. A.) [N. A.]
6	Bergau und Liebers (2015) [Deutschland]	Untersuchung pragmatisch-kommunikativer Sprachkompetenzen von Kindern in der Übergangsphase vom Elementar- zum Primarbereich	Mixed-Method Teilstudie 1, Abschnitt Kita: Quantitativ (Fähigkeitstests, Befragungen, Fragebögen) [2 Längsschnitte; 4 Erhebungszeitpunkte] {Kinder, Eltern, EuE, Einrichtungsleitungen} Teilstudie 2, Abschnitt Hort: Qualitativ (Videoaufnahme, Beobachtungsbogen) [Längsschnitt; 6 Erhebungszeitpunkte] {Kinder, EuE, pädagogische Fachkräfte}	Teilstudie 1: T1: 155 (N. A.) [Mehrsprachigkeit, Down-Syndrom, Allgemeine Entwicklungsverzögerung/altersungemäße Entwicklung in den Bereichen Sprache, Konzentration/Wahrnehmung, Motorik, Hören] Teilstudie 2: 10 (N. A.) [Sprachförderung]	Teilstudie 1: T1: 6;0 (0,3) [N. A.] T3: 6;6 (0,3) [N. A.] Teilstudie 2: N. A. (N. A.) [N. A.]
		Identifikation förderlicher Kommunikations- und Interaktionsstrategien pädagogischer Fachkräfte im Kindergarten und der Grundschule für kindliche Gesprächskompetenzen			
7	Binz et al. (2012) [Deutschland]	Untersuchung der Praktiken und Möglichkeiten zur Elternpartizipation in kooperativen Netzwerken in Kindergärten und Grundschulen	Qualitativ (Ethnographie; Interviews, Gruppendiskussionen, Inhaltsanalyse, teilnehmende Beobachtung) [Längsschnitt; 2 Erhebungszeitpunkte] {Eltern, EuE, LuL}	0 (–) [–]	–
8	Chan^a^ (2010) [Hong Kong]	Untersuchung & Beschreibung von Übergangsaktivitäten für LuL, Eltern und Kinder während des Übergangs vom Kindergarten zur Grundschule sowie deren Effektivität	Mixed-Method (Fragebögen, halbstrukturierte Interviews) [N. A.] {Eltern, LuL}; (Beobachtung, Interviews) [N. A.] {Kinder}	40 (N. A.) [N. A.]	N. A. (N. A.) [6–7]
9	Chan^a^ (2012) [Hong Kong]	Erwartungen an den Übergang in die Grundschule aus Sicht von LuL, Eltern und Kindern	Mixed-Method (Fragebögen, halbstrukturierte Interviews) [N. A.] {Eltern, EuE, LuL}; (Beobachtung) [N. A.] {Kindergartenkinder}; (Follow-Up-Interview) [N. A.] {Grundschulkinder}	72 (N. A.) [N. A.]	N. A. (N. A.) [N. A.]
		Beschreibung (a) der Erwartungen von LuL und Eltern an die kindlichen Kompetenzen in fünf für einen guten Übergang relevanten Entwicklungsbereichen (vorschulische und soziale Fähigkeiten, Eigenständigkeit, persönliche Qualitäten, Selbstdisziplin), und (b) tatsächliche Kompetenzen der Kinder in diesen Bereichen sowie Leistungen nach dem Übergang			
10	Cook et al. (2019) [USA]	Beschreibung von in Head Start Programmen verwendeten Praktiken und Prozessen zur Koordination mit Grundschulen für die Unterstützung des Übergangs in die Vorschule	Qualitativ (Interview) [N. A.] {Head-Start-Leitungspersonal}	N. A. (N. A.) [Kinder aus bildungsfernen & sozioökonomisch benachteiligten Familien/Regionen; Kinder mit Verhaltensauffälligkeiten]	N. A. (N. A.) [N. A.]
		Ableitung und Darstellung der Einstellung von Head Start Leitungen bzgl. (a) des Nutzens der Praktiken und Merkmale erfolgreicher Koordination und (b) der Herausforderungen der Koordination mit Grundschulen & Verbesserungsoptionen			
11	Correia und Marques-Pinto (2016) [Portugal]	Vergleich des Anpassungsprozesses von Kindern während des Übergangs aus Sicht der Eltern und LuL	Qualitativ (Fokusgruppeninterviews) [N. A.] {EuE, LuL, Eltern}	0 (–) [–]	–
		Untersuchung der Risikofaktoren für die Entwicklung von Anpassungsproblemen aus einer präventiven Perspektive (Perspektive des Kindes, der Familien und der Schule)			
12	Corsaro und Molinari^b^ (2000) [Italien]	Identifikation von „Priming Events“ im Kindergarten	Qualitativ (vergleichende Ethnographie; tw. Audio- und Videographie, Interviews) [Längsschnitt] {FuF, Eltern, LuL}	21 (57) [N. A.]	5,5 (N. A.) [N. A.]
		Beschreibung der Auswirkungen kollektiver Aktivitäten in den „Priming Events“ auf die Vorstellungen des Übergangs der Kinder			
		Vergleich der Vorstellungen der Eltern und der Kinder			
13	Corsaro et al.^b^ (2003) [Italien]	Freundschaftsprozesse einer Gruppe von Kindern im letzten Kindergartenjahr und während des Übergangs in die Grundschule	Qualitativ (vergleichende Ethnographie; Erfassung von Interaktions-Episoden, tw. Audio- und Videographie, Interviews) [Längsschnitt] {Eltern, Kinder, FuF}		
14	Daley et al. (2011) [USA]	Beschreibung von unterstützenden Übergangspraktiken für Kinder mit sonderpädagogischem Unterstützungsbedarf („Special Needs“) beim Übergang in die Vorschule	Quantitativ (Interviews, Fragebögen) [Längsschnitt; 5 Erhebungszeitpunkte] {N. A.}	1989 (28) [mit Förderbedarf: Sprachstörung, Entwicklungsverzögerung, Autismus, Lernstörung, geistige/emotionale/körperliche Behinderung]	6,1 (N. A.) [N. A.]
		Untersuchung des Einflusses der sozioökonomischen Lebensumstände auf die Form der Unterstützung			
15	Faust et al. (2012) [Deutschland]	Untersuchung, durch welches Modell psychosoziale Probleme beim Übergang in die Grundschule besser vorhergesagt werden	Quantitativ (Einschätzungsfragebögen) [Längsschnitt; 4 Erhebungszeitpunkte] {Eltern, LuL}	554 (28) [N. A.]	Bei Schuleintritt: 6,4 (0,4) [5,3–7,5]
		Einfluss von individuellen, familiären und institutionellen Faktoren auf die Bewältigung des Übergangs vom Kindergarten in die Grundschule			
16	Forest et al. (2004) [USA]	Identifikation & Integration kritischer Übergangselemente aus empirischer Forschung	Quantitativ (Interviews mit Ratingfragen) [N. A.] {Eltern, EuE, Vorschul-LuL}	3 (0) [Autismus]	5,7 (0,6) [5–6]
		Entwicklung eines Instruments zur Bewertung des Übergangsprozesses (für Familien, Schulen und Behörden)			
		Pilotierung im Feld			
17	Fried und Stude (2011) [Deutschland]	Untersuchung des Einflusses des häuslichen Kontextes auf die Entwicklung von Erzählkompetenzen (Nach‑, Fantasie- und Bilderzählung) von Kindern während des Übergangs vom Kindergarten in die Grundschule	Quantitativ (Einschätzungsfragebögen) [Längsschnitt; 3 Erhebungszeitpunkte] {FuF; Eltern}	382 (N. A.) [N. A.]	T1: 5,7 (0,4) T2: 6,2 (0,4) T3: 7,2 (0,4) [5–7]
18	Fröhlich et al. (2011) [Deutschland]	Wirksamkeit der Lobo-Programme (Förderung der phonologischen Bewusstheit) im Kindergarten und der Grundschule	Quantitativ (Fähigkeitstest, Intelligenztest, Migrationsfragebogen) [N. A.] {Kinder; Eltern}	501 (53) [Migrationshintergrund]	5,9 (0,4) [N. A.]
19	Giovannini et al. (2005) [Deutschland]	Veränderungen im kindlichen Verhalten, von Problemen und Lebenssituationen nach erstem Jahr an der Grundschule (Elternperspektive; Vergleich mit Perspektive der Klassen-LuL)	Quantitativ (Einschätzungsfragebögen) [Follow-Up-Messung] {Eltern, LuL}	Elternbefragung: 131 (48) [Verhaltensauffälligkeiten]	N. A. (N. A.) [N. A.]
20	Goble et al. (2017) [USA]	Transaktionsbeziehungen zwischen positiven sozialen Interaktionsfähigkeiten und schulischem Engagement und Leistungen von Kindern im Übergang Kindergarten – Grundschule	Qualitativ (Einschätzungsfragebögen, Fähigkeitstest) [Längsschnitt; 3 Erhebungszeitpunkte] {LuL, Kinder, Eltern}	241 (49) [82 % niedriger SÖS]	4;5 (0,3) [3;9–5]
21	Gower et al. (2014) [USA]	Untersuchung des Zusammenhangs zwischen physischer & relationaler Aggression im Kindergarten und der Schüler-Lehrer-Beziehung, Akzeptanz durch Gleichaltrige und internalisierende Verhaltensprobleme	Mixed-Method (Beobachtung der Kinder, Fragebögen) [N. A.] {N. A.}	190 (48) [N. A.]	4,8 (0,3) [4–5,6]
22	Höke und Arndt (2015) [Deutschland]	Untersuchung der institutionsübergreifenden Kooperation zwischen EuE und LuL im Hinblick auf Gelingensbedingungen und Kernelementen mit professionsspezifischen Unterschieden	Qualitativ (Interviews) [N. A.] {EuE, Grundschul-LuL}	0 (–) [–]	–
23	Hosokawa und Katsura (2019) [Japan]	Auswirkungen von autoritärer und permissiver Erziehung in der frühen Kindheit auf externalisierendes & internalisierendes Verhalten von Kindern während des Übergangs in die Grundschule, inkl. Geschlechtseinflüsse	Quantitativ (Fragebögen) [Längsschnitt; 2 Erhebungszeitpunkte] {Eltern}	1668 (48) [Verhaltensprobleme]	5 (N. A.) [–]
24	Iruka et al. (2014) [USA]	Übergangsprofile vom Kindergarten in die Vorschule basierend auf der Bewertung von akademischen Fähigkeiten und Sozialverhalten	Mixed-Method (Fähigkeitstests, Einschätzungsfragebogen, Interview, Beobachtung) [Längsschnitt; 4 Erhebungszeitpunkte] {FuF, Eltern, Kinderbetreuerinnen und -betreuer, LuL}	700 (0) [Afroamerikanische Jungen]	4,4 (0,4) [4–5]
		Vorhersage des Übergangsprofils aufgrund des sozioökonomischen Status, der elterlichen Erziehung und der Teilnahme an einem zentrumsbasierten Programm			
25	Kluczniok et al. (2015) [Deutschland]	Mögliche Übergangsprobleme und Einflüsse auf das Übergangserleben bezüglich kognitiver, sprachlicher, sozialer und schulischer Anforderungen (Elternperspektive)	Mixed-Method (Einschätzungsfragebögen, Kompetenztests, Beobachtungsbögen, Interview) [Längsschnitt; 7 Erhebungszeitpunkte] {Eltern, Kinder}	191 (50) [N. A.]	6,1 (0,4) [5,2–6,8]
		Zufriedenheit der Eltern mit der Übergangsgestaltung			
		Prüfung des Nutzens kooperativer Strukturen zwischen Kindergarten und Grundschule sowie der Teilnahme am KiDZ-Projekt			
26	Krajewski (2008) [Deutschland]	Zusammenhang zwischen phonologischem Bewusstsein, dem Arbeitsgedächtnis und der Mengen-Zahlen-Kompetenzen im Kindergartenalter und der Mathematik Schulleistung in der dritten Klasse	Quantitativ (Schultests) [Längsschnitt; 4 Erhebungszeitpunkte] {Kinder}	108 (51) [tw. Migrationshintergrund]	T1: 5;7 (N. A.) [4;11–6;6] T4: 8;8 (N. A.) [8;0–9;7]
27	Krompàk (2015) [Schweiz]	Rekonstruktion und Beschreibung von Selektionsprozessen und Bildungsungleichheit im pädagogischen Alltag beim Übergang vom Kindergarten in die Grundschule	Qualitativ (teilnehmende Beobachtung, in-situ-Gespräche) [Längsschnitt; ethnographisch] {Kinder}	1 (100) [Mehrsprachigkeit]	N. A. (N. A.) [N. A.]
28	Ladd und Price (1987) [USA]	Identifikation von Prädiktoren für die soziale & schulische Anpassung von Kindern an die neue Schulumgebung	Qualitativ (Beobachtung, Fragebögen, Telefoninterview, Einschätzungsfragebögen, Interview) [Längsschnitt; 3 Erhebungszeitpunkte] {Beobachterinnen und Beobachter, Eltern, LuL, Interviewerinnen und Interviewer}	T1: 63 (46) [N. A.]	5 (0,4) [N. A.]
29	Lee und Goh (2012) [Singapur]	Beschreibung eines Ansatzes zur Adressierung von Problemen beim Übergang in die Grundschule	Qualitativ (Interviews, Beobachtungen) [N. A.] {EuE = FuF, Kinder}	14 (36) [N. A.]	N. A. (N. A.) [5–6]
30	Li und Lau (2019) [China]	Beitrag von Konflikten zwischen LuL und SuS im Kindergarten zur schulischen Anpassung des Kindes in der Grundschule	Quantitativ (Einschätzungsfragebögen, Selbstregulationsfähigkeitstest) [Längsschnitt; 2 Erhebungszeitpunkte] {EuE, Eltern, Kinder, LuL}	T1: 324 (52) [N. A.] T2: 247 (51) [N. A.]	T1: 5,9 (0,3) [N. A.]
		Mediierender Effekt der Selbstregulierung und moderierender Effekt positiver elterlicher Beziehungen mit anderen			
31	Lichtblau (2014) [Deutschland]	Einflüsse der Mikrosysteme Familie, Kindergarten und Schule auf die Interessenentwicklung der Kinder	Mixed-Method (leitfadengestützte Interviews, soziokultureller Fragebogen, episodisch-narratives Interview, Einschätzungsfragebögen, Intelligenztest) [Längsschnitt; 3 Erhebungszeitpunkte] {Kinder, Eltern, EuE, LuL}	13 (46) [soziokulturell benachteiligt]	5;5 (N. A.) [N. A.]
32	LoCasale-Crouch et al. (2008) [USA]	Einsatz von Übergangspraktiken im Kindergarten durch EuE	Quantitativ (Beobachtung, Einschätzungsfragebögen) [N. A.] {Beobachterinnen und Beobachter, LuL, EuE}	722 (51) [soziokulturell benachteiligt]	N. A. (N. A.) [N. A.]
		Zusammenhang zwischen Praktiken und Einschätzungen der EuE bzgl. sozialer, selbstregulatorischer und akademischer Fähigkeiten der Kinder beim Start der Vorschule			
33	Margetts und Phatudi (2013) [Südafrika]	Verständnis und Praktiken von Schulleiterinnen und Schulleitern, LuL, Eltern und Kindern bzgl des Übergangs in die Grundschule	Qualitative Fallstudie (semi-strukturierte Interviews in Fokusgruppen) [N. A.] {Direktorinnen und Direktoren, LuL, Eltern, SuS}	12 (N. A.) [niedrigerer SÖS und wirtschaftlich benachteiligte Region, v. a. dunkelhäutige Einwohner]	N. A. (N. A.) [N. A.]
34	McWayne et al. (2009) [USA]	Untersuchung des akademischen und sozialen Funktionsniveaus von Kindern aus einkommensschwachen Familien während des Übergangs von der Vorschule in die erste Klasse	Quantitativ (Fragebögen) [Längsschnitt; 2 Erhebungszeitpunkte] {LuL, EuE, Grund-SuS}	152 (48) [Kinder aus einkommensschwachen Familien]	7,2 (0,3) [N. A.]
35	Petermann et al. (2008) [Deutschland]	Adaption der „Lehrereinschätzliste für Sozial- und Lernverhalten (LSL)“ für den Kindergartenbereich	Quantitativ (Einschätzungsfragebögen) [N. A.] {EuE}	91 (47) [LRS]	6,2 (0,5) [5–7]
36	Probst (2009) [Deutschland]	Sprachliche Förderung von neu eingeschulten Kindern mit geringen phonologischen Kompetenzen inkl. Analyse des Nutzens	Quantitativ (Fähigkeitstest) [N. A] {Kinder}	T1: 163 (50) [LRS] T2: 187 (50) [LRS]	~ 6^c^ (N. A.) [N. A.]
37	Rietveld (2008) [Australien]	Analyse von Fallstudien hinsichtlich von Kontexteinflüssen (v. a. auf Mikroebene des Kindes) auf Übergange von Kindern mit und ohne Down-Syndrom	Qualitativ (Beobachtung während kontinuierlicher narrativer Erzählungen) [N. A.] {Kinder}; (Interviews) [N. A.] {Eltern, LuL, Peers}	4 (0) [2 Kinder mit Down-Syndrom]	4;11 (N. A.) [N. A.]
38	Roncancio-Moreno und Branco (2017) [Brasilien]	Ko-Konstruktion von Kindern bzgl. ihrer Entwicklungsprozesse im Übergang vom Kindergarten in die Grundschule	Qualitativ (Interviews, Beobachtungen, semistrukturelle Aufgaben, Journaleinträge der Kinder) [Längsschnitt; 2 Erhebungszeitpunkte] {Kinder, Eltern, LuL, Peers}	3 (66) [N. A.]	5 (N. A.) [N. A.]
39	Rule et al. (1990) [USA]	Entwicklung und Evaluation eines Curriculums zur Vermittlung sog. Allgemeiner Überlebensfähigkeiten im Kindergarten/in der ersten Klasse	Qualitativ (Literatursuche, Beobachtung) [N. A.] {Kinder, LuL, FuF}	Teilstudie 1: 20 (N. A.) [N. A.] Teilstudie 2: 18 (28) [15 Kinder mit Behinderung]	Teilstudie 1: N. A. (N. A.) [N. A.] Teilstudie 2: N. A. (N. A.) [4;4–4;10]
40	Salmi und Kumpulainen (2019) [Finnland]	Charakterisierende Motive des kindlichen Erlebens beim Schuleintritt	Qualitativ (Zeichnungen, aufgezeichnete Interviews, ethnographische Feldnotizen) [N. A.] {FuF, Kinder}	19 (N. A.) [N. A.]	N. A. (N. A.) [6–7]
		Vereinbarkeit der kindlichen Motive mit den subjektiven Anforderungen der Grundschule			
41	Schmitt et al. (2017) [USA]	Untersuchung des Zusammenhangs zwischen Wohnsitzmobilität während des Übergangs in die Vorschule und externalisierenden und internalisierenden Verhaltensproblemen bei Kindern	Mixed-Method (Einschätzungsfragebögen, Interview, Fragebogen) [N. A.] {LuL, Eltern/Betreuerinnen und Betreuer}	300 (53) [Kinder aus einkommensschwachen Familien in nicht-elterlicher Fürsorge]	4 (0,6) [2,9–5,5]
		Untersuchung eines moderierenden Effekts familienunterstützender Dienstleistungen			
42	Schulting et al. (2005) [USA]	Effekte schulbasierter Kindergartenübergangsrichtlinien und -praktiken auf akademische Leistungen der Kinder	Mixed-Method (Fähigkeitstests, Interviews, Fragebögen) [Längsschnitt; 2 Erhebungszeitpunkte] {Kinder, Eltern, LuL, Schulverwalterinnen und -verwalter}	17.212 (49) [N. A.]	5;7 (0,4) [5,3–6,2]
		Einfluss des elterlichen Engagements			
43	Seven (2010) [Türkei]	Zusammenhang von Bindungsrepräsentation und Sozialverhalten von Kindern mit deren schulischer Anpassung beim Übergang in die Grundschule	Mixed-Method (Einschätzungsfragebogen, Geschichtenerzählung mit Puppe) [Längsschnitt; 4 Erhebungszeitpunkte] {Grund-SuS, Eltern, LuL, FuF}	80 (43) [N. A.]	6,8 (N. A.) [N. A.]
44	Stenseng et al. (2015) [Norwegen]	Langfristige Auswirkungen von sozialer Ausgrenzung im Kindergarten auf die kindliche Selbstregulation nach dem Übergang in die Grundschule	Mixed-Method (Fragebogen) [N. A.] {LuL}; (Fragebogen, Interview) [Längsschnitt; 2 Erhebungszeitpunkte] {Eltern}	762 (50) [N. A.]	N. A. (N. A.) [4–6]
		Prüfung einer wechselseitigen Beziehung zwischen sozialer Ausgrenzung und der Entwicklung von Selbstregulation inkl. Geschlechtseffekte			
45	Thieme (2012) [Deutschland]	Anwendung des bewegungs- und ressourcenorientierten Konzeptes „Bewegung macht stark für die Schule“	Quantitativ (standardisiertes Puppeninterview) [N. A.] {Kinder}	229 (N. A.) [N. A.]	N. A. (N. A.) [N. A.]
		Stärkung des Selbstkonzeptes bei der Transition vom Kindergarten zur Grundschule durch gemeinsame Bewegungsaktivitäten von Vor- und Grundschulkindern			
46	Wildenger und McIntyre (2011) [USA]	Untersuchung der Elternperspektive auf den Übergang vom Kindergarten zur Schule	Quantitativ (Fragebögen) [N. A.] {Eltern}	86 (41) [N. A.]	5,4 (0,3) [N. A.]
		Fokus auf die Sorgen und Bedürfnisse sowie die Involvierung in die Vorbereitung der Eltern			
47	Winter und Panagiotopoulou (2017) [Deutschland]	Rekonstruktion von Differenzierungsprozessen zwischen Schulkindern und Kindern vor der Einschulung	Quantitativ (Beobachtungsprotokolle, in-situ-Gespräche) [ethnographisch] {FuF, LuL}	N. A. (N. A.) [Inklusions-SuS]	N. A. (N. A.) [N. A.]

*N.* *A.* keine Information vorhanden, *LRS* Lese-Rechtschreib-Schwäche, *SÖS* sozioökonomischer Status, *T* *[Ziffer]* Testzeitpunkt, *LuL* Lehrerinnen und Lehrer, *EuE* Erzieherinnen und Erzieher, *FuF* Forscherinnen und Forscher, *SuS* Schülerinnen und Schüler

^a^Gleiche Stichprobe, in Chan (2012) wurden zusätzlich *n* = 32 Erstklässlerinnen und Erstklässler aufgenommen

^b^Gleiche Stichprobe

^c^Bis auf wenige Ausnahmen haben alle Kinder das siebte Lebensjahr begonnen

### Erhebungsländer

**Figure 1:**
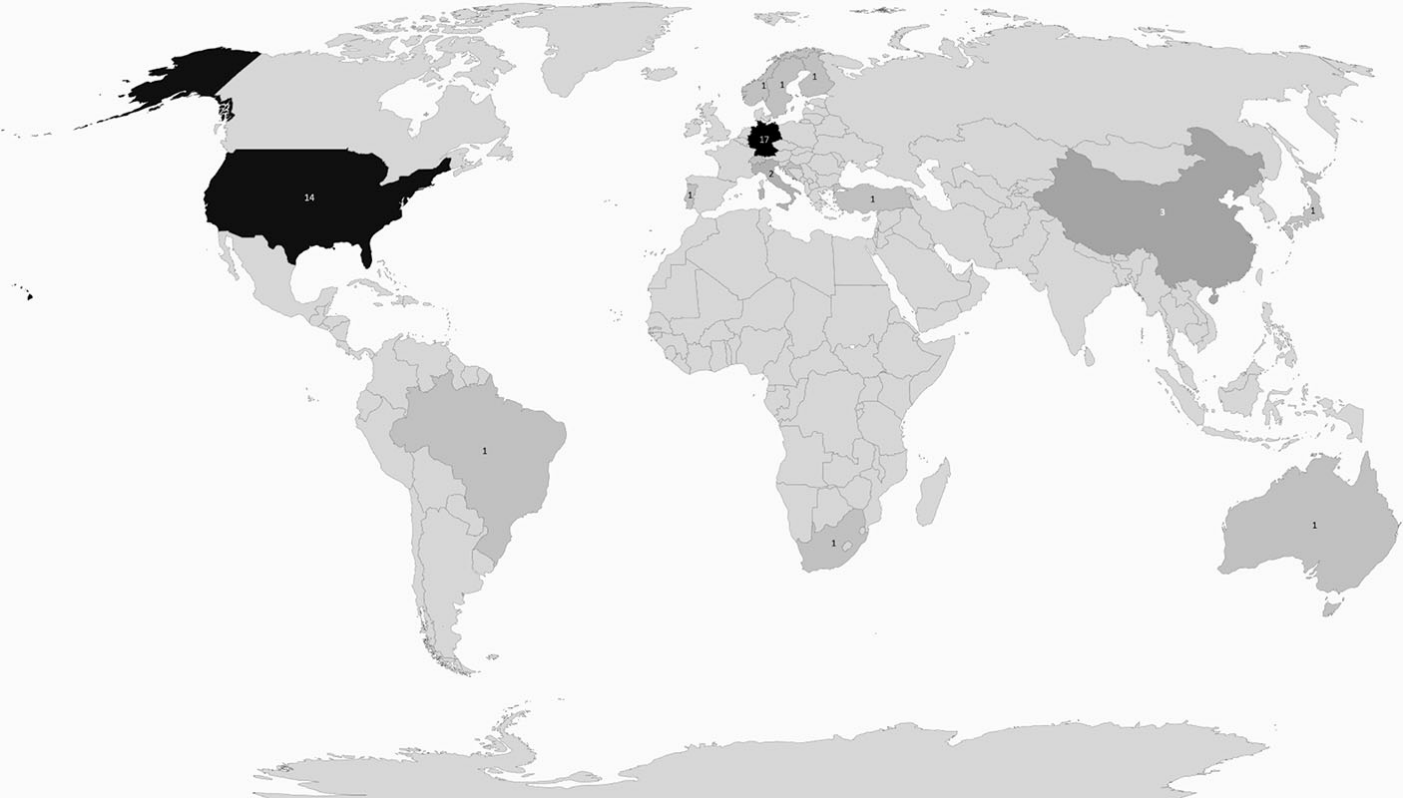

Die Mehrzahl der Studien wurden in Deutschland (*k* = 16; 33 %) und den USA (*k* = 13; 29 %) durchgeführt (siehe Abb. 1). In *k* = 2 Studien wurden Daten in China erhoben; die übrigen *k* = 13 Studien wurden in unterschiedlichen Ländern durchgeführt.

### Jahrgänge

Die eingeschlossenen Studien wurden von 1987 bis 2019 publiziert. Während für den Publikationszeitraum zwischen 1990 und 2000 keine Studien identifiziert werden konnten, zeichnet sich ab der Jahrtausendwende eine Zunahme des Forschungsinteresses am Übergang zwischen Kindergarten und Grundschule ab. Ab 2008 wurden jährlich mindestens zwei Studien publiziert. Die größte Publikationsrate wurde 2017 mit *k* = 6 Veröffentlichungen gefunden.

### Fachzeitschriften

Die eingeschlossenen Studien wurden in 35 unterschiedlichen Fachzeitschriften publiziert. Insgesamt 13 Fachzeitschriften waren deutsch- und 22 englischsprachig. Die meisten Studien wurden mit jeweils *k* = 3 Publikationen in den Fachzeitschriften *Early Childhood Research Quarterly *und *Early Child Development and Care* veröffentlicht.

### Studiendesign und Datenerhebung

**Figure 2:**
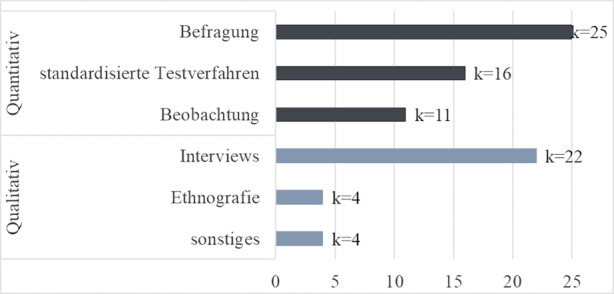

Im Großteil der Studien (*k* = 39) wurde das jeweilige Forschungsziel explorativ untersucht. Von den eingeschlossenen Studien wiesen *k* = 18 ein qualitatives Design auf. Quantitative Studiendesigns wurden in *k* = 16 Studien bzw. Mixed-Methods-Designs in *k* = 13 Studien angewendet (s. Tab. 1). Die eingesetzten Methoden zur Datenerhebung umfassten bei quantitativen (Teil‑)Studiendesigns insbesondere Befragungen, standardisierte Testverfahren (z. B. Test für Phonologische Bewusstheitsfähigkeiten (TPB; Fricke und Schäfer 2008) in der Studie von Fröhlich et al. (2011) oder der *Peabody Picture Vocabulary Test III* (PPVT-III) und der *Woodcock-Johnson Test of Achievement III* (WJ-III) in der Studie von Goble et al. (2017)) und Beobachtungen. Qualitative (Teil‑)Studiendesigns setzten vorwiegend Interviews zur Datenerhebung ein; vereinzelt wurden ethnographische oder andere Methoden (z. B. Geschichtenerzählung, Journaleinträge, Action-Research-Ansätze) umgesetzt (s. Abb. 2).

**Figure 3:**
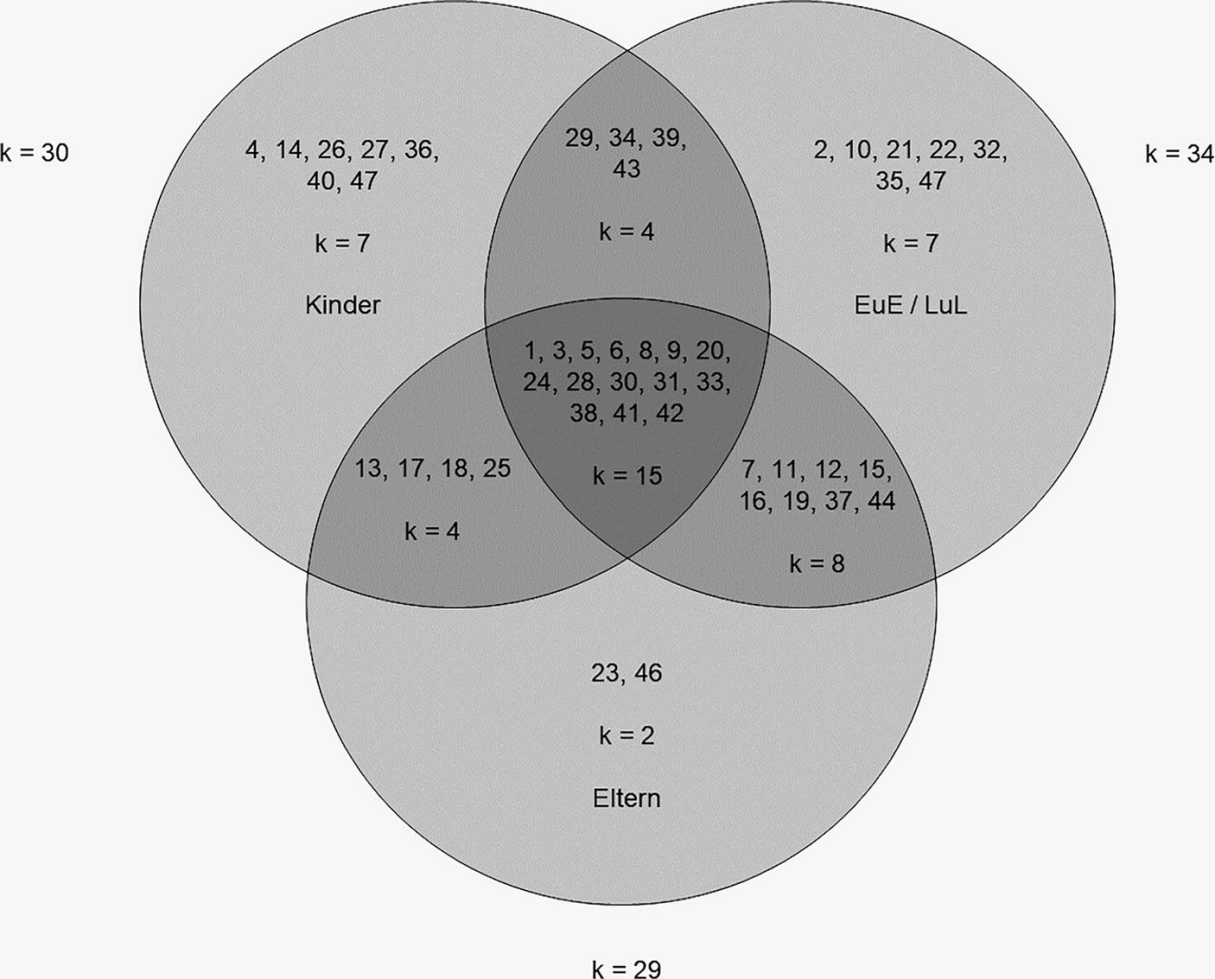

Bei der Datenerhebung wurden die Perspektiven von Kindern (*k* = 30), deren Eltern (*k* = 29) sowie von Professionellen (d. h. Erzieherinnen und Erzieher oder Lehrerinnen und Lehrer; *k* = 34) berücksichtigt (s. Abb. 3).

### Forschungsziele und Outcomes

Der Großteil der eingeschlossenen Studien zielte auf die Exploration unterschiedlicher Ursachen und Zusammenhänge im Kontext der Transition ins formale Schulsystem ab. Sechs Studien untersuchten die Effektivität von Interventionsmaßnahmen im Rahmen von schulischen Übergängen (s. Tab. 1). Zwei Studien explorierten sowohl das Feld und bewerteten eine Intervention (Benner et al. 2017; Kluczniok et al. 2015). In den Studien von Forest et al. (2004) und Petermann et al. (2008) wurde jeweils ein Diagnostikinstrument zur Bewertung des Übergangs durch Behörden, Schulen und Familien bzw. zur Erfassung des Sozial- und Lernverhaltens vor dem Schuleintritt entwickelt. Insbesondere folgende Outcomes wurden im Rahmen der eingeschlossenen Studien in den Blick genommen:

- Schulische Fähigkeit am Übergang (*k* = 11),
- Übergangsbewältigung (*k* = 10),
- Persönlichkeitsmerkmale (*k* = 5) und
- Verhaltens- und Entwicklungsauffälligkeiten (*k* = 4).

In zwei Studien wurde zudem die externe Unterstützung am Übergang, z. B. im Sinne der Elternpartizipation, betrachtet. Zur Operationalisierung der Outcomes wurden entweder kindbezogene (*k* = 16), akteursbezogene (*k* = 9) oder strukturelle Merkmale der Bildungssysteme (*k* = 11) erfasst.

### Untersuchte Stichproben

Als Stichprobe wurde im Rahmen des vorliegenden Beitrages jeweils die Gruppe der Kinder am Übergang zwischen Kindergarten und Grundschule verstanden, wobei in einigen Studien keine Kinder, sondern ausschließlich die Perspektive von z. B. Eltern oder Fachkräften untersucht wurden (s. Tab. 1). Die Stichprobengröße in den eingeschlossenen quantitativen Studien rangierte zwischen *n* = 3 und *n* = 1989 Kindern (*M* = 450,2; *SD* = 558,0) mit einer Gesamtstichprobe von *N* = 7203 Kindern. Der Anteil von Mädchen lag in den eingeschlossenen quantitativen Studien zwischen 0 und 53 % (*M* = 42,7 %; *SD* = 14,3 %). Die Stichprobengröße in den qualitativen Studien rangierte zwischen *n* = 0 und* n* = 241 Kindern (*M* = 40,3; *SD* = 68,7) mit einer Gesamtstichprobe von *N* = 645 Kindern. Der Anteil von Mädchen in den qualitativen Studien lag zwischen 0 und 100 % (*M* = 51,3; *SD* = 24,6). Die Stichprobengröße in den identifizierten Mixed-Methods-Studien rangierte zwischen *n* = 0 und *n* = 17.212 Kindern (*M* = 2045,4; *SD* = 4767,8) mit einer Gesamtstichprobe von *N* = 24.545 Kindern. Der Anteil von Mädchen lag in den eingeschlossenen Mixed-Methods-Studien zwischen 0 und 50 % (*M* = 42,6 %; *SD* = 16,4 %). Die Kinder waren zum Zeitpunkt der Datenerhebung in den *k* = 26 Studien (54 %), die Angaben zum Alter der untersuchten Kinder enthielten, zwischen 2,9 und 7,5 Jahre alt (*M* = 5,7; *SD* = 0,7), s. Tab. 1. Fokussiert wurden in *k* = 11 der eingeschlossenen Studien Kinder mit sonderpädagogischem oder zusätzlichem Förderbedarf bzw. in *k* = 9 Studien sozial marginalisierte Kinder, z. B. aus niedrigen sozioökonomischen Systemen und wirtschaftlich benachteiligten Regionen (s. Tab. 1).

### Übergangsbezogene Forschungsschwerpunkte und zentrale Ergebnisse

**Figure 4:**
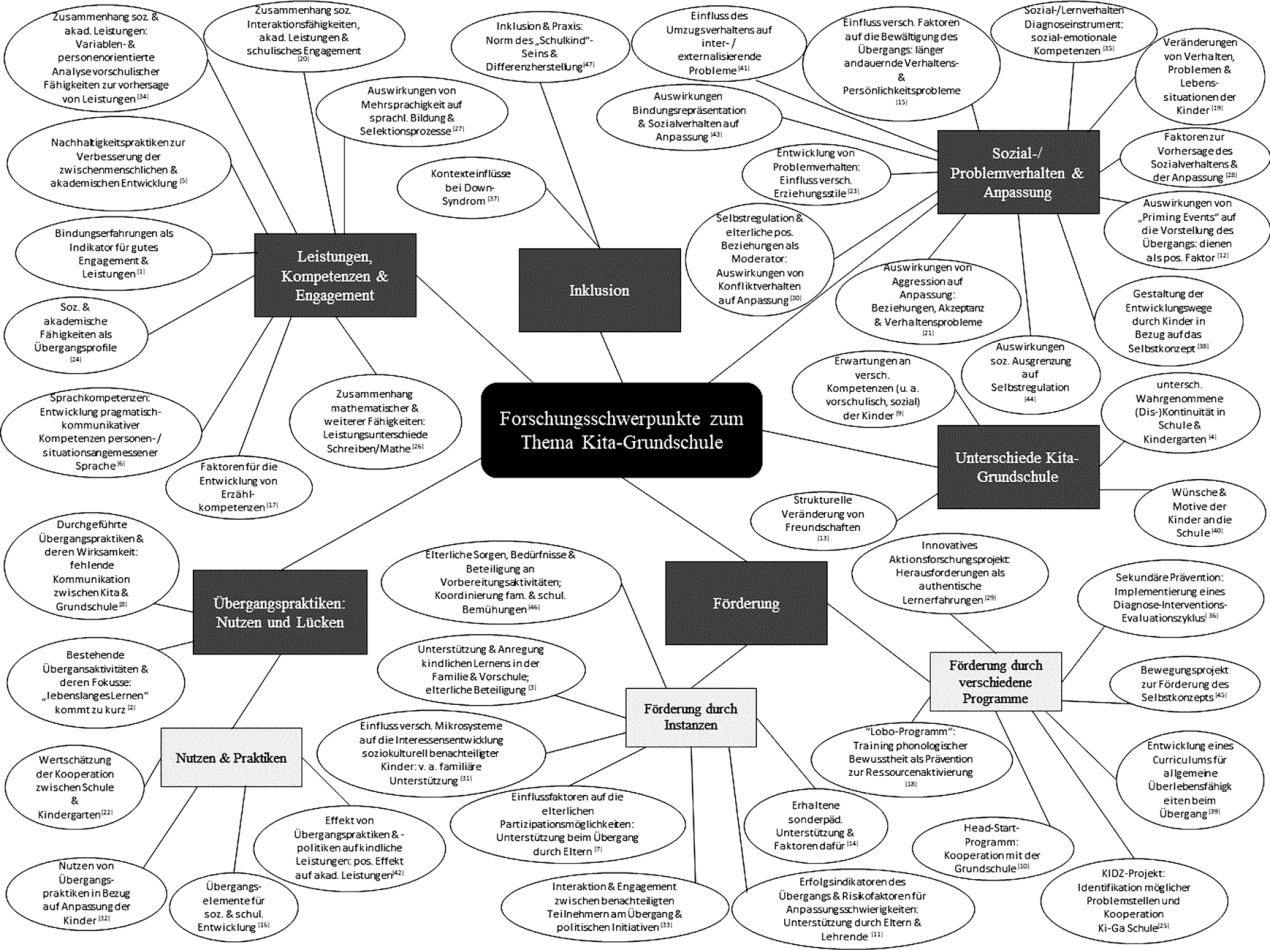

Inhaltlich lassen sich die eingeschlossenen Studien sechs übergangsbezogenen Forschungsschwerpunkten zuordnen, die häufig auch die emotionale und soziale Entwicklung der Kinder genauer in den Blick genommen haben:

- Sozial‑, Problemverhalten und Anpassung am Übergang,
- Unterschiede zwischen Kindergarten und Grundschule,
- Leistungen, Kompetenzen und Engagement,
- Inklusion,
- Förderung während des Übergangs sowie
- Übergangspraktiken (s. Abb. 4).

#### Sozialverhalten, Problemverhalten und Anpassung am Übergang

Insgesamt *k* = 9 Studien beschäftigten sich mit Sozial- bzw. Problemverhalten und Anpassung von Kindern am Übergang zwischen Kindergarten und Grundschule. Giovannini et al. (2005) beschrieben, dass Lehrerinnen und Lehrer sowie Eltern unterschiedliche Veränderungen in Bezug auf das Verhalten, Probleme und Lebenssituationen der Kinder feststellen (z. B. Zunahme oppositionellen Verhaltens oder Konzentrationsprobleme, aber auch Zunahme der Selbständigkeit), obwohl sich laut Child Behavior Checklist keine signifikanten Veränderungen abzeichneten. Problembehaftete Transitionen wurden auf länger andauernde Verhaltensprobleme und Auffälligkeiten im Temperament der Kinder zurückgeführt, sodass möglichst frühe Interventionen empfohlen wurden (Faust et al. 2011). Um Förderbedarfe bereits vor Schuleintritt zu erkennen und Verhaltensproblemen vorzubeugen, entwickelten Petermann et al. (2008) eine „Lehrereinschätzliste für Sozial- und Lernverhalten (LSL)“ als ressourcenorientiertes Verfahren, das schulfähigkeitsrelevante sozial-emotionale Fähigkeiten (z. B. Selbstkontrolle, differenziertes Lernverhalten) erfasst und auf den Kindergartenbereich übertragbar ist. Die Analyse von Roncancio-Moreno und Branco (2017) zeigte, dass Kinder ihre eigenen Entwicklungswege ko-konstruieren, indem sie aktiv die kulturellen Anregungen von bedeutenden sozialen Anderen verinnerlichen. Zeigen Kinder in der Vorschule vermehrt körperlich aggressives Verhalten, sagte dies eine geringere Akzeptanz durch Gleichaltrige und vermehrte Konflikte mit Erzieherinnen und Erziehern bzw. Grundschullehrerinnen und Grundschullehrern voraus (Gower et al. 2014). Ladd und Price (1987) zeigten, dass aggressive Kinder von ihren Peers eher abgelehnt und feindselig eingeschätzt werden als kooperative Kinder. Aggressives und hyperaktives Verhalten wurde im Zusammenhang einer autoritären Erziehung in der Kindheit gefunden (Hosokawa und Katsura 2019). Mädchen, Kinder mit Vorkenntnissen, ältere Kinder sowie Kinder aus Elternhäusern mit höheren Bildungsabschlüssen hatten einen besseren Schulstart (Faust et al. 2011). Auf struktureller System- und Interventionsebene wurde gezeigt, dass frühe Erfahrungen in der Vorschule die Anpassung an neue Regeln, Zeitpläne und Teilnehmerinnen- und Teilnehmerstrukturen im Unterricht im Sinne von „Priming Events“ begünstigen (Corsaro und Molinari 2000). Stenseng et al. (2015) zeigten, dass soziale Ausgrenzung eine beeinträchtigte Entwicklung der dispositionellen Selbstregulierung vorhersagte, während eine schlechte Selbstregulierung eine verstärkte soziale Ausgrenzung vorhersagte. Soziale Ausgrenzung beeinträchtigte die Entwicklung der Selbstregulierungsfähigkeiten der Kinder, wobei eine schlechte Selbstregulierung die Wahrscheinlichkeit der Ausgrenzung erhöhte. Für Konflikte zwischen Lehrerinnen oder Lehrern und Schülerinnen oder Schülern hingegen wurde ein negativer Zusammenhang mit der Selbstregulation der Kinder gefunden, die wiederum die spätere Anpassung an das Schulsystem vorhersagte (Li und Lau 2019). Mehrfache Umzüge während der Übergangsphase waren signifikant assoziiert mit einer stärkeren Externalisierung und Internalisierung von Verhaltensproblemen (Schmitt et al. 2017).

#### Unterschiede zwischen Kindergarten und Grundschule

Insgesamt *k* = 4 Studien untersuchten system- und personenbezogene Unterschiede zwischen Kindergarten und Grundschule (s. Abb. 4). Generell wurde der Kindergarten in den Studien aus der Perspektive der Kinder als Ort gesehen, an dem sie betreut werden, spielen können und auf die Schule vorbereitet werden; die Schule hingegen diene dem Lernen und sei geprägt von der Benotung (Babić 2017). Chan (2012) identifizierte unterschiedliche Erwartungen bei der Bewertung der für einen erfolgreichen Übergang notwendigen Fertigkeiten durch Eltern, Erzieherinnen und Erziehern sowie Lehrerinnen und Lehrer. Auf personenbezogener Ebene zeigte sich, dass sich Freundschaften im Kindergarten – anders als in der Grundschule – kaum vom Status oder dem Geschlecht der Kinder beeinflussen lassen (Corsaro et al. 2003). Wünsche und Motive von Kindern an das System Schule sowie den Übergang hingen mit Möglichkeiten, sich körperlich zu bewegen, zu spielen, Beziehungen aufzubauen und den sich verändernden Rollen und Identitäten zusammen (Salmi und Kumpulainen 2019).

#### Leistungen, Kompetenzen und Engagement

Potenziell relevante Aspekte für die Leistungen, Kompetenzen und das Engagement von Kindern bereits vor dem Übergang wurden in *k* = 9 Studien untersucht (s. Abb. 4). Eine sichere Bindung zur Mutter sowie die Unterstützung des Explorationsverhaltens durch Erzieherinnen und Erzieher prägten die Lernfreude, Selbstmotivation und Anstrengungsbereitschaft von Kindern (Ahnert und Harwardt 2008). Bergau und Liebers (2015) fanden, dass sich die pragmatisch-kommunikativen Sprachkompetenzen von Kindern am Übergang im letzten Vorschulhalbjahr signifikant verbessern. Insbesondere vorschulische Kompetenzen waren relevant für die späteren schulischen Leistungen (McWayne et al. 2009). Zudem konnten Leistungsunterschiede in Rechtschreibung und Mathematik am Ende der ersten Klasse auf unterschiedliches phonologisches Bewusstsein und die visuell-räumliche Komponente des Arbeitsgedächtnisses zurückgeführt werden (Krajewski und Schneider 2009). Sogenannte Nachhaltigkeitspraktiken (z. B. gleichbleibende Lernumgebungen in kleinen Klassen, hochqualifizierte Lehrerinnen und Lehrer, ausreichend Lernzeit, Übergangshilfen wie Orientierungstage und Programminformationen), gemäß dem amerikanischen PK‑3^2^ Modell zur Förderung von Kontinuität und Konsistenz über den Übergang hinweg, können unabhängig von schulischen oder familiären Selektionsmechanismen Lesezuwächse und zwischenmenschliche Fähigkeiten (z. B. Teilen, Trost spenden) nach dem Übergang begünstigen (Benner et al. 2017). Fried und Stude (2011) untersuchten die Entwicklung der Erzählkompetenzen wie Nach‑, Fantasie- und Bilderzählungen während des Übergangs und zeigten die Bedeutung der elterlichen Unterstützung für die Entwicklung auf. Iruka et al. (2014) beleuchteten Übergangsprofile afroamerikanischer Jungen und den Einfluss sozioökonomischer Faktoren und fanden im Familieneinkommen und im Erziehungsverhalten der Eltern (z. B. emotionale Unterstützung) Einflussfaktoren auf die Entwicklung der akademischen Leistungen. Krompàk (2015) fand für die Schweiz, dass Bildungsungleichheiten aufgrund von Selektionsentscheidungen auf der Grundlage von Schuleignungstests entstehen, die Aspekte wie Schulreife und Instruktionsverständnis, jedoch weniger die globale Sprachkompetenz in den Blick nehmen.

#### Inklusion

Das Thema Inklusion wurde explizit in *k* = 2 Studien aufgegriffen. Die in den Studien veröffentlichten Befunde deuten darauf hin, dass selbst in inklusiven Schulen ein Selektionsprozess innerhalb oder außerhalb des Unterrichts stattfand, bei dem normativ zwischen „richtige[n]“ und „noch nicht richtigen“ Schulkindern unterschieden wurde (Winter und Panagiotopoulou 2017, S. 27). Insgesamt wurde die Inklusion bzw. Exklusion bei Kindern mit Down-Syndrom eher kontextuell (z. B. Art der Beziehung zu Erzieherinnen und Erziehern sowie Lehrerinnen und Lehrern) als durch Kompetenzbeschreibungen (z. B. im Bereich der emotionalen und sozialen Entwicklung oder der Motorik) in den Blick genommen (Rietveld 2008).

#### Förderung während des Übergangs

Insgesamt *k* = 14 Studien untersuchten die Förderung der Kinder am Übergang vom Kindergarten zur Grundschule. In *k* = 7 Studien wurden verschiedene Programme untersucht, die überwiegend auf die Förderung von Merkmalen und Kompetenzen der Kinder abzielen. Untersucht wurde beispielsweise das Lobo-Programm zur Förderung der phonologischen Bewusstheit von Kindergarten- und Schulkindern, das präventiv zur Ressourcenaktivierung dienen soll (Fröhlich et al. 2011). Thieme (2012) beschrieb zielgerichtete ressourcenorientierte Bewegungsanlässe im Kontext Schule zur Förderung des physischen, schulischen und sozialen Selbstkonzepts von Vorschulkindern. Gezeigt wurde, dass Herausforderungen verwandelt in authentische Lernerfahrungen dazu beitragen können, dass sich schon vor dem Übergang eine positive Einstellung zur Schule entwickelt und sich potenzielle Ursachen von Angst und Stress für Kinder und Eltern reduzieren (Lee und Goh 2012). Rule et al. (1990) identifizierten im Rahmen einer Curriculums-Entwicklung, dass Kinder sogenannte Überlebensfähigkeiten (z. B. unabhängig arbeiten, an Gruppen teilnehmen) aufweisen müssen, um den Übergang in die erste Klasse erfolgreich zu bewältigen. Probst (2009) zeigte, dass Kinder mit Defiziten in der phonologischen Bewusstheit sich mit Hilfe eines entsprechenden Trainingsprogramms in ihren Fähigkeiten signifikant den Leistungen ihrer Mitschülerinnen und Mitschülern annähern konnten. Dieses Trainingsprogramm sollte eine Sekundärprävention darstellen, bei der ein Diagnose-Interventions-Evaluationszyklus implementiert wird. Kluczniok et al. (2015) konnten zeigen, dass kindbezogene Merkmale (z. B. Rechenfähigkeiten) relevanter sind als die Kooperation zwischen Kindergarten und Schule, die mittels Head-Start-Programmen gefördert werden kann. Weitere sechs Studien (Arndt et al. 2013; Binz et al. 2012; Correia und Marques-Pinto 2016; Lichtblau 2014; Margetts und Phatudi 2013; Wildenger und McIntyre 2011) belegten, dass die Übergangsbewältigung nicht nur durch spezielle Programme, sondern auch durch die am Übergang beteiligten Systeme (v. a. Unterstützung in der Familie) positiv beeinflusst werden kann. So fand Lichtblau (2014) beispielsweise Hinweise darauf, dass sich die Interessen einzelner Kinder infolge differenter familiärer Unterstützungsbedingungen unterschiedlich entwickeln und durch soziokulturelle Belastungssituationen moderiert werden können.

#### Übergangspraktiken

Nutzen und Lücken von Übergangspraktiken, also institutionsübergreifende Kooperationen zur Transitionsgestaltung, betrachteten *k* = 6 Studien. Alatalo et al. (2016) diskutieren, dass ein kontinuierliches, systemverbindendes Lernen für Kinder durch ein mangelndes Professionsverständnis für Erzieherinnen und Erzieher seitens der aufnehmenden Schule sowie Lehrerinnen und Lehrern eingeschränkt wird. Zwischen Kindergarten und Grundschule fehlte es laut Chan (2010) an professionellen Kommunikationsstrukturen. Höke und Arndt (2015) beschreiben die Bedeutung von gegenseitiger Wertschätzung in der Kooperation von Kindergarten und Grundschule. Hinsichtlich des Nutzens von Übergangspraktiken zeigte sich, dass sich diese positiv auf die Übergangsbewältigung hinsichtlich Anpassungs- und Leistungsfähigkeit auswirken können (LoCasale-Crouch et al. 2008; Schulting et al. 2005). Forest et al. (2004) arbeiteten Elemente für einen erfolgreichen Transitionsplan heraus, die am Übergang autistischer Kinder in die Grundschule untersucht wurden.

## Diskussion

Der vorliegende Beitrag hatte zum Ziel, den nationalen und internationalen empirischen Forschungsstand zum Übergang vom Kindergarten in die Grundschule von Kindern abzubilden und zusammenzufassen. Die Ergebnisse bilden ein wachsendes Forschungsinteresse an diesem Themengebiet seit der Jahrtausendwende und insbesondere in den letzten 13 Jahren ab. Insgesamt ist die Menge an relevanten Studien, die mittels des Scoping-Verfahrens identifiziert werde konnte, vor dem Hintergrund einer internationalen Suche über mehrere Jahrzehnte allerdings als gering einzuschätzen. Die Forschung zum Übergang zwischen Kindergarten und Grundschule scheint zudem stark westlich geprägt zu sein. So wurden kaum Studien aus Nicht-Industrieländern oder der nicht-westlichen Welt identifiziert. Forschungsschwerpunkte zum Übergang zwischen Kindergarten und Grundschule wurden in den vergangenen Jahren insbesondere in den Bereichen der Übergangsförderung (z. B. mithilfe von Förderprogrammen), des Sozial- und Problemverhaltens bzw. der Anpassung von Kindern sowie Einflussfaktoren auf deren Kompetenzen, Leistungen und Engagement in der Übergangsphase in die Grundschule, wie auch institutioneller Praktiken zur Übergangsgestaltung (z. B. Kooperation und Kommunikation zwischen Kindergarten und Grundschule) gesetzt.

Studien, die spezifisch emotionale und Verhaltensauffälligkeiten (z. B. einen (prognostizierbaren) Förderbedarf im Bereich der emotionalen und sozialen Entwicklung, psychische Erkrankungen) infolge nicht oder nicht gut bewältigter Übergänge untersuchten, wurden jedoch nicht identifiziert. Mit Blick auf die vielfältigen psychosozialen Folgen eines nicht bzw. schlecht bewältigten Übergangs in die Grundschule scheint an dieser Stelle ein Forschungsdesiderat hinsichtlich systemverbindender und/oder inklusiver Konzepte zu bestehen, die besonders auf benachteiligte Kinder abzielen und zur frühestmöglichen Prävention von Verhaltensauffälligkeiten oder anderen Beeinträchtigungen beitragen können. Auf die Bedeutung gezielter vorschulischer Förderung in den Bereichen der emotionalen und sozialen Kompetenz gerade in der Post-Pandemie-Phase weisen aktuelle Studienergebnisse u. a. von Mays et al. (2023) hin. Hier konnten mittlere bis starke negative Auswirkungen während der Covid-19-Schließungen auf die Entwicklung grundlegender sozial-emotionaler Kompetenzen bei Vorschulkindern dokumentiert werden. Diese Ergebnisse sind zum einen vergleichbar mit den ganz aktuellen Ergebnissen der Elternbefragungen von Bantel et al. (2021), Raw et al. (2021) und zum anderen auch mit den Schlussfolgerungen aus erst vor Kurzem publizierten qualitativen Interviews mit Vorschulkindern (Duran 2021; Gramigna und Poletti 2020). Ferner bestätigen sie auch die Ergebnisse von Steinmayr et al. (2022).

In Folgestudien sollte somit geklärt werden, ob eine zusätzliche Förderung sozial-emotionaler Kompetenzen durch strukturierte, aber alltagstaugliche, spielorientierte und dennoch modularisierte Angebote wie „Löwenstark“ (Mays et al. 2022) hier hilfreiche Anregungen für eine verstärkt binnendifferenzierte, sozial-emotionale vorschulische Bildungsarbeit in sozialen Brennpunkten grundsätzlich geben, ob diese mit Blick auf die Covid-19 Pandemie eine kompensatorische Wirkung entfalten können und ob eine gezieltere vorschulische Förderung emotionaler und sozialer Kompetenz einen Beitrag zu einer gesteigerten Anschlussfähigkeit – insbesondere für benachteiligte Kinder aus Risikofamilien – leisten kann.

Trotz einer Kategorisierbarkeit der empirischen Erkenntnisse in die weiteren genannten Forschungsschwerpunkte fällt zudem ein hohes Maß an Heterogenität z. B. hinsichtlich der Themen- und Zielsetzungen, Fragestellungen und untersuchten Outcomes auf. Auch zwischen Studien innerhalb eines Forschungsschwerpunktes existieren – über die grundsätzliche Schwerpunktsetzung hinaus – wenig verbindende Aspekte, vielmehr stehen sie häufig mit geringem gegenseitigem Bezug nebeneinander. Zudem wurden bisher kaum Langzeitstudien oder Replikationen bzw. Fortführungen von Studien durchgeführt, sondern häufig beobachtend-explorativ geforscht. Eine zentrale Herausforderung bei der Zusammenfassung internationaler Forschungsergebnisse stellt dabei die Unterschiedlichkeit der Schulsysteme bzw. der Systeme vorschulischer Förderung dar. Oftmals ist dadurch eine ungeprüfte Übertragung der „Evidenz“ auf das deutsche System nicht zulässig.

Die methodische Qualität der identifizierten Studien ist außerdem als eher gering einzuschätzen. Obwohl die vorliegenden Ergebnisse also die große Bandbreite relevanter Informationen und Befunde für den Übergang vom Kindergarten in die Grundschule verdeutlichen, können aktuell wenig generalisierende Aussagen oder Aussagen über größere Zusammenhänge getroffen werden.

### Limitationen und Stärken

Bei der Interpretation der Ergebnisse müssen eine Reihe von Limitationen beachtet werden. Zum einen bildet der vorliegende Beitrag aufgrund der gewählten Einschlusskriterien nicht die gesamte Forschungsliteratur ab. Zum anderen müssen methodische Limitationen bedacht werden: Ein Bias der Reviewerinnern und Reviewer bei der Datenbanksuche, der Definition der Einschlusskriterien sowie im Selektionsprozess (d. h. Screening und Bewertung der Volltexte) ist nicht auszuschließen. Ebenso ist ein Publikations-Bias hinsichtlich signifikanter Befunde trotz Strategien, diesen zu verringern, nicht auszuschließen.

Nichtsdestotrotz erlaubt der vorliegende Beitrag durch die Differenzierung von bereits untersuchten Forschungsfeldern, eingesetzten Methoden und empirischen Befunden eine Orientierung im Forschungsfeld zum Übergang zwischen Kindergarten und Grundschule. Die aktuellen Ergebnisse können zudem hilfreich sein für die Entwicklung und empirische Untersuchung weiterer Forschungsfragen, ein besseres Verständnis der Herausforderungen und Ressourcen am Übergang zwischen Kindergarten und Grundschule sowie für die (Weiter‑)Entwicklung von Interventionen bzw. Förderprogrammen oder von Übergangspraktiken zur Unterstützung der betroffenen Kinder und deren Familien.

## Footnotes

1 Dieser Begriff wurde gewählt, um auch Studien aus anderen Ländern einschließen zu können, in denen der Übergang vergleichsweise früh eintritt (z. B. USA: Übergang vom Kindergarten in die Vorschule).

2 Die amerikanische PreK-3-Bewegung, die sich auf den Zeitraum zwischen Vorschule und dritter Klasse bezieht, will die frühe Bildung mithilfe unterschiedlicher verbundener Initiativen revolutionieren (Graves 2006). Dazu gehören der allgemeine Zugang zu kostenloser öffentlicher Vorschule, ein verpflichtender Ganztagskindergarten und ein Lehrplan, der nahtlos von der Vorschule bis zur dritten Klasse reicht. Die stärkere Einbeziehung der Eltern ist ebenfalls ein wichtiger Schwerpunkt.
